# Cigarette Smoking Triggers Colitis by IFN-γ^+^ CD4^+^ T Cells

**DOI:** 10.3389/fimmu.2017.01344

**Published:** 2017-10-31

**Authors:** Gihyun Lee, Kyoung-Hwa Jung, Dasom Shin, Chanju Lee, Woogyeong Kim, Sujin Lee, Jinju Kim, Hyunsu Bae

**Affiliations:** ^1^Department of Science in Korean Medicine, Kyung Hee University, Seoul, South Korea; ^2^Department of Korean Physiology, College of Pharmacy, Kyung Hee University, Seoul, South Korea

**Keywords:** cigarette smoke, Crohn’s disease, IFN-γ, Th1, colitis

## Abstract

The increased incidence of Crohn’s disease in smokers has been recently reported, suggesting a strong association of cigarette smoke (CS) with colitis. However, the mechanism of the action of CS on colitis has not yet been explored. Here, we demonstrate that CS exposure is sufficient to induce colitis in mice. Interestingly, the colitis is mainly mediated by Th1, but not Th17, responses. CD4^+^ T-cell depletion or T-bet/IFN-γ deficiency protects against the development of colitis induced by CS. Additionally, IFN-γ-producing CD4^+^ T cells play a substantial role in CS-induced colitis. The adoptive transfer (AT) of effector T cells from CS-exposed WT mice into colitis-prone mice caused these mice to develop colitis, while the AT of effector T cells from IFN-γ knock-out mice did not. These findings have implications for broadening our understanding of CS-induced pathology and for the development of novel therapeutic strategies to treat Crohn’s disease.

## Introduction

Cigarette smoking is blamed for the death of approximately six million people globally every year (1). Although the knowledge of the risks posed by cigarette smoking has been well documented, the worldwide prevalence of cigarette consumption is estimated to be in excess of one billion persons (2, 3). Studies have implicated cigarette smoking as a significant risk factor for a number of chronic disorders, such as respiratory (4), cerebrovascular (5), and cardiovascular diseases (6, 7). To date, there are increasing numbers of reports suggesting a link between cigarette smoking and colitis. Cigarette smoke (CS) has been established as the most robust risk factor for Crohn’s disease (8, 9), and the rates of Crohn’s disease incidence are significantly increased in people with airway diseases (10). Despite these clinical and epidemiological observations that link CS exposure with Crohn’s disease, a few experimental investigations have been undertaken to explore the role of CS in intestinal homeostasis and the underlying mechanisms mediating the effects of CS on Crohn’s disease still remain unclear.

IFN-γ, a prototypical Th1/Tc1 cytokine, plays a pivotal role in the regulation of various immune responses, including leukocyte trafficking, microbicidal effector activation, pathogen recognition, antigen presentation, and cellular proliferation (11, 12). However, the overexpression of this weighty cytokine is involved in many gastric diseases, including autoimmune gastritis (13), celiac disease (14), and Crohn’s disease (15), as well as pulmonary inflammation (16).

To investigate the mechanisms mediating the effects of CS exposure on colitis, we examined whether CS exposure can induce colitis as well as lung inflammation. As expected, CS exposure caused lung inflammation-mediated Th1 and Th17 responses. Notably, CS exposure was enough to induce colitis and this colitis was mainly mediated by the Th1 response, not the Th17 response. We validated the role of the Th1 response in CS exposure-induced colitis using specific CD4^+^/8^+^ T-cell depletion or IFN-γ/T-bet knock-out mice. Finally, we confirmed that IFN-γ plays a substantial role in colitis induced by lung draining lymph node CD4^+^CD25^−^ T-cell adoptive transfer (AT) in colitis-prone mice.

## Materials and Methods

### Mice

C57BL/6J WT, IFN-γ^−/−^ (B6.129S7-Ifng^tm1Ts^/J), IL-10^−/−^ (B6.129P2-Il10^tm1Cgn^/J), and CRISPR/Cas9 knock-in (B6J.129 (Cg)-Gt (ROSA) 26Sortm1.1^(CAG-cas9*,-EGFP) Fezh^/J: WT^EGFP^) mice were purchased from the Jackson Laboratory (Bar Harbor, ME, USA). T-bet^−/−^ (Tbox21) mice were provided by Dr. Laurie Glimcher (Department of Immunology and Infection Diseases, Harvard School of Public Health). All mice were housed in cages maintained under pathogen-free conditions with air conditioning, a 12 h light and 12 h dark cycle and were provided with food and water *ad libitum*. The study was approved by the Kyung Hee University animal care and use committee. All of the experiments were performed in accordance with the approved animal protocols and guidelines established by Kyung Hee University [KHUASP (SE)-12-015]. All experiments used females that were 7–9 weeks old.

### CS Exposure

This study was performed using previously described experimental procedures, which expose mice to mainstream of CS (17, 18). The mice were exposed to the smoke from five consecutive non-filtered cigarettes (Reference 3R4F without filter; University of Kentucky, Lexington, KY, USA) four times a day, 6 days per week, using a smoking apparatus described by previously (18). Female C57BL/6J WT, T-bet^−/−^, IFN-γ^−/−^, and WT^EGFP^ mice were exposed to CS for 4 weeks, and CD4^+^ and CD8^+^ depletion, immune cell profiling in lamina propria (LP), hemoccult test and A4B7^+^ cell analysis studies were performed under 2 week CS exposure. All mice were sacrificed 24 h after the last CS exposure.

### Cytometric Bead Array (CBA) Assay

The ascending part of the colon was homogenized with a protease inhibitor cocktail (Roche Diagnostics, Mannheim, Germany) and centrifuged at 10,000 × *g* for 15 min at 4°C. The colon homogenates were assayed for Th1, Th2, and Th17 using a CBA (BD Sciences, San Diego, CA, USA) using a BD FACSCalibur flow cytometer (BD Sciences).

### Enzyme-Linked Immunosorbent Assay (ELISA)

The bronchoalveolar lavage (BAL) fluid was immediately collected after the mice were sacrificed. The lungs were lavaged and pooled three times with 1 ml of ice-cold PBS. The obtained BAL fluid was centrifuged at 3,000 × *g* for 10 min at 4°C, and the supernatants (BAL fluid) stored at −80°C. The Th1 cytokines, IFN-γ and TNF-α, were measured using a quantitative sandwich ELISA kit (IFN-γ, and TNF-α; BD Sciences) according to the manufacturer’s protocol. The optical density was measured at 450 nm using a microplate reader (SoftMax Pro software; Sunnyvale, CA, USA). The optical densities obtained for IFN-γ and TNF-α were each divided by the total protein concentrations of the respective BAL fluid samples for standardization purposes. The total protein concentrations were determined using a Bio-Rad protein assay (Bio-Rad, Hercules, CA, USA) according to the manufacturer’s protocol.

### Quantitative Real-time PCR (qRT-PCR) Assay

Total RNA was prepared from frozen colon tissue homogenates with an easy-BLUE™ RNA extraction kit (iNtRON Biotech., Sungnam, Republic of Korea). The cDNA synthesis was carried out for at 42°C and 5 min at 94°C using a cDNA synthesis kit (Bioneer Corporation., Daejeon, Republic of Korea). qRT-PCR for IFN-γ and TNF-α was performed with a SYBR Green I master mix using a Lightcycler^®^ 480 system (Roche, Basel, Switzerland) as previously described in Jung et al. (19). The IFN-γ, TNF-α, and β-actin genes were amplified using the following primers: IFN-γ: forward (F), 5′-TCA AGT GCG ATA GAT GTG GAA GAA-3′ and reverse (R), 5′-TGG CTC TGC AGG ATT TTC ATG-3′, TNF-α: F, 5′-CAT CTT CTC AAA ATT CGA GTG ACA A-3′ and R, 5′-TGG GAG TAG ACA AGG TAC AAC CC-3′, and β-actin: F, 5′-AGA GGG AAA TCG GTG AC-3′ and R, 5′-CAA TAG TGA CCT GGC GCT-3′. IFN-γ and TNF-α expressions were normalized to β-actin expression (20).

### BAL Cell Analysis

To perform BAL fluid collection, the mice were sacrificed and a tracheal cannula was slowly inserted. Three times via the tracheal cannula, 1 ml of ice-cold PBS was delivered and recovered by gentle manual aspiration. The collected BAL fluid was centrifuged at 3,000 × *g* for 10 min at 4°C, and the cell pellet was washed and finally resuspended in 1 ml of PBS. First, the total viable cells in the resulting pellet were counted using a trypan blue stain. To count the differential cells (neutrophils, macrophages, and lymphocytes), BAL fluid cells were adhered to glass slides using Cytospin (Sandon, Waltham, MA, USA) with Diff-Quick staining (Life Technologies., Auckland, New Zealand). The stained BAL cell slides were mounted with Canada balsam (Showa Chemical Co. Ltd., Tokyo, Japan). The BAL cells were counted under a light microscope as we previously reported (19, 21). The result was indicated as the cell number × 10^4^.

### Flow Cytometer (FACS) Analysis

The mesenteric lymph nodes (MLNs) were disrupted over a wire mesh screen. The colonic LP was isolated into a single-cell suspension as previously described in Bosurgi et al. (22). The lung was dissociated into a single-cell suspension using a mouse lung dissociation kit (Miltenyi Biotec, Bergisch Gladbach, Germany) with the gentle MACS™ dissociator, according to the manufacturer’s protocol. The red blood cells were lysed in BD Pharm Lyse™ lysing solution (BD Sciences). The single cells were stimulated in RPMI 1640 supplemented with 10% fetal bovine serum, 50 UI/ml penicillin, and 50 µg/ml streptomycin (Hyclone, Logan, UT, USA) for 5 h with 50 ng/ml PMA/1 μg/ml Ionomycin (Sigma-Aldrich, St. Louis, MO, USA), respectively, in the presence of 0.66 µl/ml BD Golgistop™ protein transport inhibitor (BD Sciences). Intracellular IFN-γ and surface marker CD4 were assessed using a Mouse Th1/Th2/Th17 Phenotyping Kit (BD Sciences) following the manufacturer’s instructions. The stimulated cells were incubated with the following antibodies: CD4-FITC, IFN-γ-PE, and IL-17A-APC (e-Bioscience, San Diego, CA, USA). To examine production of IFN-γ in CD4, CD8, or NK cells, splenocytes were stimulated for 5 h with PMA, Ionomycin, and BD Golgistop™. The cells fixed and stained with cell surface marker CD4-PE, CD8-APC, or NK1.1-FITC (e-Bioscience). Then, intracellular IFN-γ was stained. To elucidate the effect of CS exposure on immune cell population in colonic LP, single cells from colonic LP were stained with the following antibodies: B220-PE, CD4-FITC, CD4-APC, CD8-APC, CD11b-APC, CD25-PE, CD45-FITC, F480-FITC, and Gr1-PE (e-Bioscience). To examine an expression of A4B7, single cells from blood and spleen were stained with CD4-APC and A4B7-PE (e-Bioscience) antibodies. All of the sample data were acquired by a FACSCalibur flow cytometer using Cell Quest Pro software (BD Sciences) and generated in graphical and tabular formats using FlowJo software (Tree Star Inc., Ashland, OR, USA).

### CD4^+^ T-Cell and CD8^+^ T-Cell Depletion

mAbs were used for the *in vivo* depletion of CD4^+^ and CD8^+^ T-cell subsets. The C57BL/6J WT mice were injected once with 500 µg i.p. antimouse CD4 Abs (clone 2.43), CD8 Abs (clone GK1.5), or rat IgG (Sigma-Aldrich) every 3 days from days 3 to 11, in 3-day intervals. The efficacy of CD4^+^ T-cell and/or CD8^+^ T-cell depletion was analyzed using a FACSCalibur flow cytometer.

### Purified CD4^+^CD25^−^ T Cells AT

CD4^+^CD25^−^ T cells were highly purified from the lung draining lymph nodes of WT and IFN-γ^−/−^ mice using the CD4^+^CD25^+^ T cells Treg isolation kit and magnetic bead separation (Miltenyi Biotec). The purity of the isolated cells was analyzed by flow cytometry prior to AT (>88% of the CD4^+^ cells were CD25^−^). The isolated CD4^+^CD25^−^ T cells (5 × 10^5^/mouse) were suspended in 200 µl PBS and injected via i.v. into IL-10^−/−^ mice. After 14 days of the AT, the mice were sacrificed.

### Histologic Analysis

The mice were transcardially perfused with a saline solution containing 0.5% sodium nitrate and heparin (10 U/ml). The lungs and colons were removed from the perfused mice, fixed with 10% neutral-buffered formalin (IMEB Inc., San Marcos, CA, USA), and embedded in paraffin. A section of the lung (4-µm thick) and transverse colons (7-µm thick) from each mouse were stained with hematoxylin and eosin and visualized on an Olympus BX51 microscope (Olympus, Tokyo, Japan) equipped with a DP71 digital camera (Olympus). A total histological score of the lung was calculated for each mouse as described previously (18). Briefly, the lung sections were scored from 0 to 5 by readers according to the following criteria: 0 = normal; 1 = very mild; 2 = mild; 3 = moderate; 4 = marked; 5 = severe inflammation. The lung sections were examined under 200× magnifications to quantify the mean alveolar airspace (MAA). MAA was a quantitative assessment of lung structure that was determined by dividing the sum of the alveolar airspace areas divided by the number of identified alveoli using Image Pro-Plus 5.1 software (Media Cybernetics, Inc., Silver Spring, MD, USA). The colon sections were assigned a score from 0 to 3 as described previously (23). The histological analyses were performed in a blinded fashion.

### Statistical Analysis

All of the values are presented as the mean ± SEM. The statistical significance (*p* < 0.05 for all analyses) was assessed using two-tailed Student’s *t*-test for single comparisons or by two-way ANOVA with repeated measures using Prism 5.01 software (Graphpad Software Inc., San Diego, CA, USA).

## Results

### Chronic CS Exposure Is Sufficient for the Induction of Th1-Mediated Colitis

**Figure 1:**
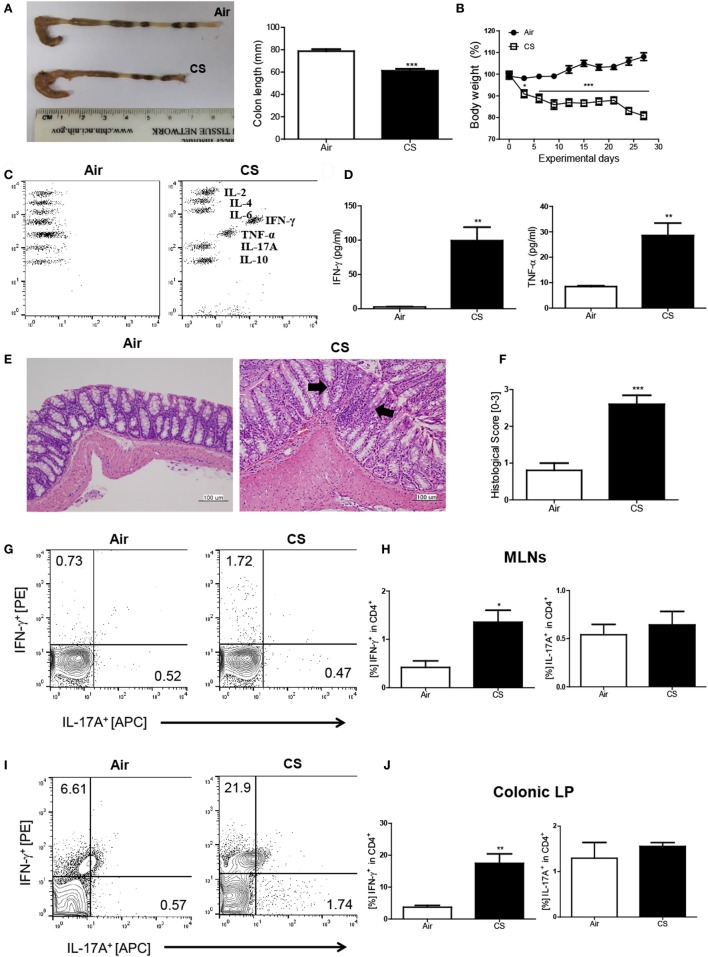
Cigarette smoke (CS) exposure-induced colitis. **(A)** Representative images of whole colons (each group) and colon length (*n* = 9–10). **(B)** Body weight changes during CS exposure (*n* = 9–10). **(C,D)** The concentration of IL-2, IL-4, IL-6, IFN-γ, TNF-α, Il-17A, and IL-10 in ascending colon tissue (*n* = 5). **(E)** To observe the infiltration of inflammatory cells, mucosal thickness, and loss of cryptal cells, the colon specimens were fixed, embedded in paraffin, sectioned, and stained with hematoxylin and eosin (magnification 200×). **(F)** The black arrows indicate submucosal and mucosal thickness. The colon histological score was calculated by a 5-point score system (*n* = 5). **(G)** The representative flow cytometric plots of IFN-γ^+^ and IL-17A^+^ in CD4^+^ T cells in the mesenteric lymph nodes. **(H)** The populations of IFN-γ^+^ and IL-17A^+^ cells in CD4^+^ T cells (*n* = 5). **(I)** The representative flow cytometric plots of IFN-γ^+^ and IL-17A^+^ on CD4^+^ T cells in the colonic lamina propria (*n* = 5). **(J)** The populations of IFN-γ^+^ and IL-17A^+^ cells in CD4^+^ T cells (*n* = 5). Data are shown as the means ± SEM, and *p* value was estimated by unpaired *t* test (***p < 0.001, **p < 0.01, and *p < 0.05 vs. air).

First, we wanted to examine whether CS exposure could induce Crohn’s disease-like illness. Therefore, we exposed the mice to CS as described in a previous study (18) and evaluated the impact of CS exposure in the bowel. The colon length was significantly shorter (air group: 77.1 ± 2.3 mm vs. CS group: 56.8 ± 0.8 mm, Figure 1A) and the body weight was markedly reduced (Figure 1B) by CS exposure after 4 weeks of CS exposure. Histologic analysis confirmed that CS exposure significantly increased the infiltration of inflammatory cells in the submucosa, epithelial cell hyperplasia, and mucus thickness in the ascending colon (Figures 1E,F). The immune cell subset analysis in the LP demonstrated that the neutrophils and macrophages were predominantly increased upon CS exposure (Figure S1A in Supplementary Material). In addition, the fecal occult blood test showed that CS exposure for 2 weeks was enough to cause bleeding (Figure S1B in Supplementary Material). These results indicated that CS exposure could cause colitis. Next, we wanted to know whether CS exposure-induced colitis resembles Crohn’s disease (Th1/Th17-dominant colitis) or ulcerative colitis (Th2 dominant colitis). We evaluated cytokine production in inflamed regions in the bowel and found that the production of Th1 cytokines such as IFN-γ and TNF-α was highly increased (37.4- and 3.1-fold higher, respectively), while the production of none of the Th2/17 cytokines, including IL-4 and IL-17A, was increased, indicating that CS exposure-induced colitis resembles Crohn’s disease (Figures 1C,D). To confirm the Th1/17 responses in CS exposure-induced colitis, we analyzed the intracellular expression of IFN-γ and IL-17A on CD4^+^ T cells in MLNs and colonic LP. The results demonstrated that CS exposure significantly increased Th1 but not Th17 in both MLNs and colonic LP, suggesting that CS exposure specifically induces Th1-mediated bowel inflammation (Figures 1G–J).

### Chronic CS Exposure Induces Lung Inflammation

**Figure 2:**
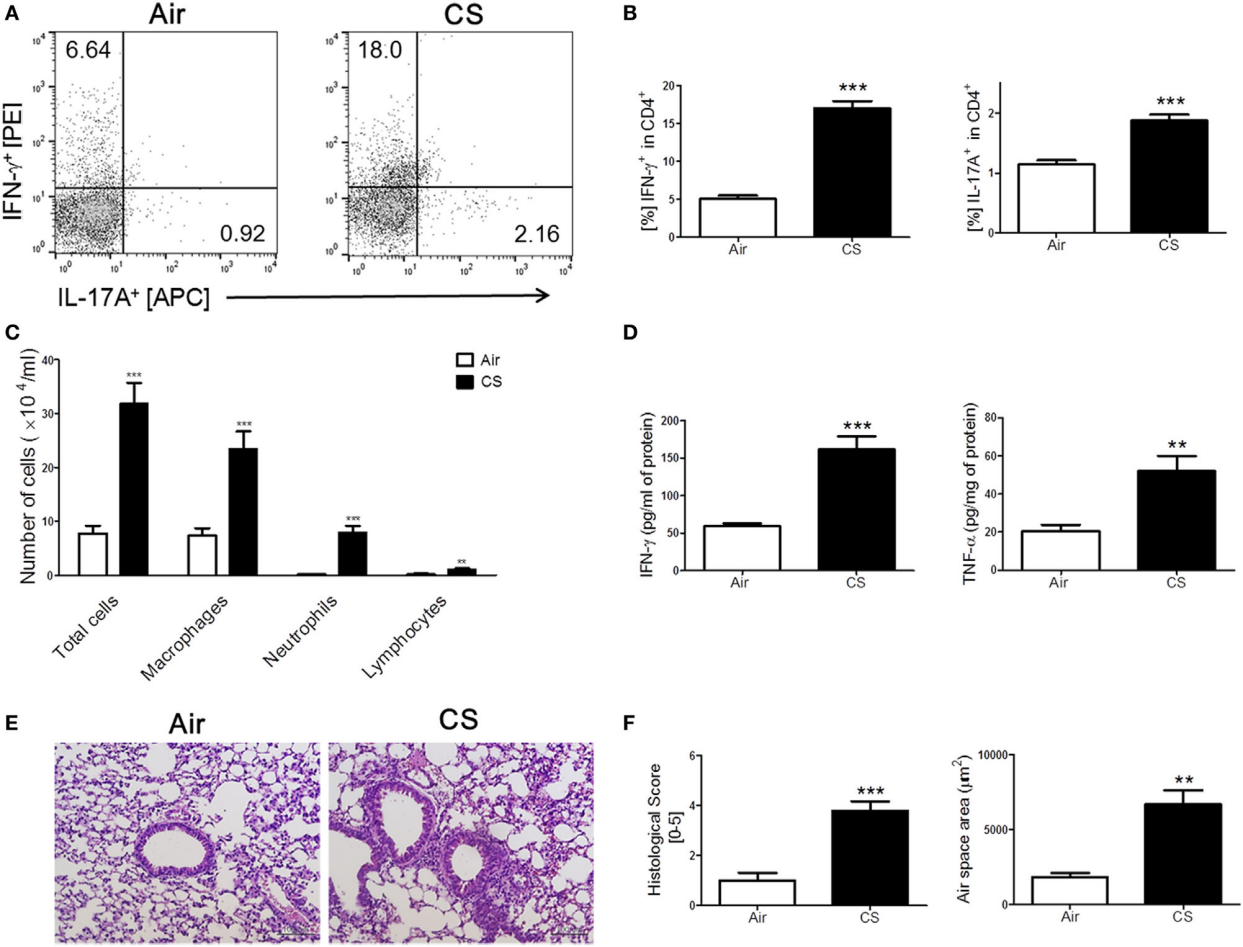
Cigarette smoke (CS) exposure-induced chronic lung inflammation. The mice were exposed to CS for a period of 4 weeks. **(A)** The representative flow cytometric plots of IFN-γ^+^ and IL-17A^+^ on CD4^+^ T cells in the lung. **(B)** The populations of IFN-γ^+^ and IL-17A^+^ cells in CD4^+^ T cells (*n* = 5). **(C)** The total cells, neutrophils, macrophages, and lymphocytes in the bronchoalveolar lavage (BAL) fluid from the lung of mice were counted using Diff-Quick staining (*n* = 9–10). **(D)** The concentrations of the Th1 cytokines IFN-γ and TNF-α in the BAL fluid were determined by enzyme-linked immunosorbent assay (*n* = 5). **(E)** The lung tissue sections were subjected to hematoxylin and eosin staining to assess histological changes and mean alveolar airspace (MAA) (*n* = 5). Assessment of lung histological change severity (left panel) was quantified using a 5-point score system. **(F)** MAA (right panel) was assessed using Image Pro-Plus 6 software based on histology (*n* = 5). Room air-exposed mice; air- and CS-exposed mice; CS. Data are shown as the mean ± SEM, and *p* value was estimated by unpaired *t* test (***p < 0.001 and **p < 0.01 vs. air).

We have repeatedly shown that CS exposure develops lung inflammation in mice (18, 24). As expected, the mice with CS exposure-induced colitis also showed signs of lung inflammation. A massive recruitment of inflammatory cells was observed in the entire lung, especially around the bronchial and peribronchial layers in the CS-exposed mice (Figure 2C). Likewise, alveolar wall destruction was also observed in their lung tissue, which resulted in enlarged airspaces (Figures 2E,F). Infiltrated immune cells reflecting pathological changes in lung parenchyma (25) were also markedly increased in the CS exposure group compared to the air exposure group (Figure 2E). We attempted to determine the involvement of Th1 and Th17 responses in lung inflammation induced by CS exposure because the Th1 response, but not the Th17 response, was observed in colonic inflammation as triggered by the same stimuli. Interestingly, the results showed that CS exposure activated both Th1 and Th17 responses in the lung, unlike in the bowel (Figures 2A,B,D).

### CS Exposure-Induced Colitis and Lung Inflammation Are Mediated Mainly by CD4^+^ T Cells

**Figure 3:**
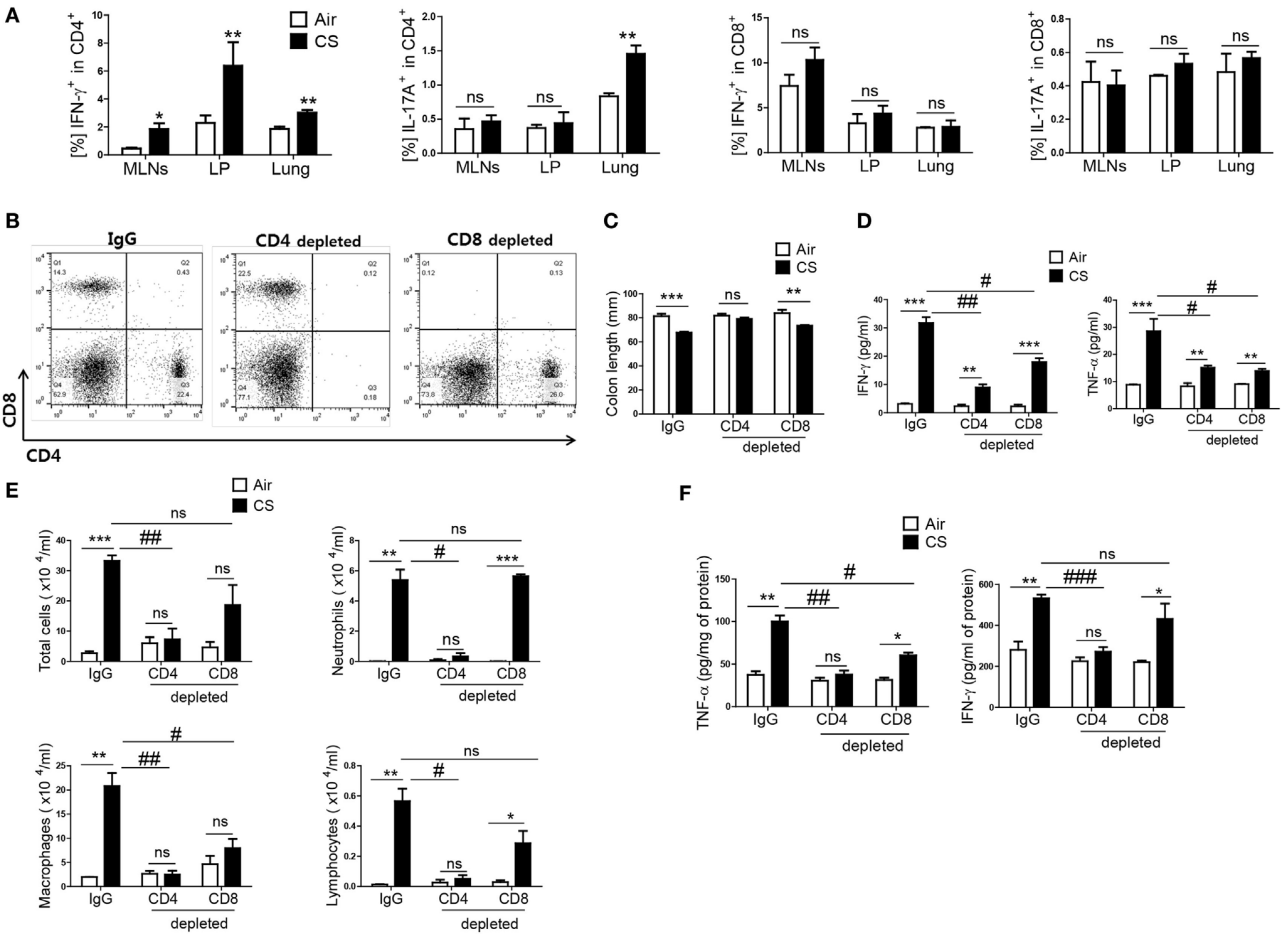
Cigarette smoke (CS) exposure-induced colitis and lung inflammation mediated by Th1 cells in CD4^+^ T cells. The mice were exposed to CS for period for 2 weeks. **(A)** Th1 and Th17 cells were analyzed by flow cytometry (gated on CD4^+^ and CD8^+^ T cells, respectively). The mice received anti-CD4 and anti-CD8 mAb to deplete CD4^+^ or CD8^+^ T cells, and anti-rat IgG was used as an isotype control. **(B)** Depletion of T-cell subsets (CD4^+^ and CD8^+^ T cells) was confirmed by flow cytometry. **(C)** The colon length was measured, and **(D)** the concentration of IFN-γ and TNF-α were evaluated in ascending colon tissue homogenate using Th1, 2, and 17 cytometric bead array. **(E)** The total cells, neutrophils, macrophages, and lymphocytes in the bronchoalveolar lavage (BAL) fluid from the lungs of mice were counted using Diff-Quick staining. **(F)** The concentrations of the Th1 cytokines IFN-γ and TNF-α in the BAL fluid were determined by using enzyme-linked immunosorbent assay. Data are shown as the mean ± SEM, and *p* value was estimated by unpaired *t* test (***p < 0.001, **p < 0.01, and *p < 0.05, ^###^*p* < 0.001, ^##^*p* < 0.01, and ^#^*p* < 0.05, and not significant; ns).

As IFN-γ and IL-17A are secreted both in CD4^+^ T cells and CD8^+^ T cells (26), we wanted to know which T-cell subtype mediated the CS exposure-induced inflammation in the colon and lung. When mice were exposed to CS, a significantly increased IFN-γ^+^CD4^+^ T-cell population was observed in the MLNs, colonic LP, and lung, but there was no significant increase in the IFN-γ^+^CD8^+^ T-cell population (Figure 3A). In addition, IFN-γ producing in CD4^+^ T cells in splenocytes was significantly increased upon CS exposure, not in CD8^+^ T and NK cells (Figure S1C in Supplementary Material). In the case of IL-17A^+^-secreting T cells, there was a significant increase in IL-17A^+^CD4^+^ T cells in the lung but not in the colon. There was no difference in IL-17A^+^CD8^+^ T cells in the lung and colon (Figure 3A). To identify specific T-cell subsets involved in CS exposure-induced colitis, we depleted CD4^+^ T cells or CD8^+^ T cells prior to CS exposure (Figure 3B). CD4^+^ T cell depletion neutralized the effect of CS exposure on colonic length. It also strongly blocked the increase in IFN-γ and TNF-α expression in the lung and the immune cell infiltration into the lung by CS exposure. In the colon, however, CD4^+^ T-cell depletion could not completely inhibit the increased expression of IFN-γ and TNF-α (Figures 3C–F). Although CD8^+^ T-cell depletion also affected colon length and the concentrations of IFN-γ and TNF-α expression in colon, the effect of CD4^+^ T-cell depletion was superior to that of CD8^+^ T-cell depletion in both the colon and lung. These results indicate that CD4^+^ T cells are the major cell population that mediates CS exposure-induced inflammation in the colon and lung, although both CD4^+^/8^+^ T cell subsets are involved.

### T-bet Deficiency Protects against the Development of Colitis and Chronic Lung Inflammation Induced by CS Exposure

**Figure 4:**
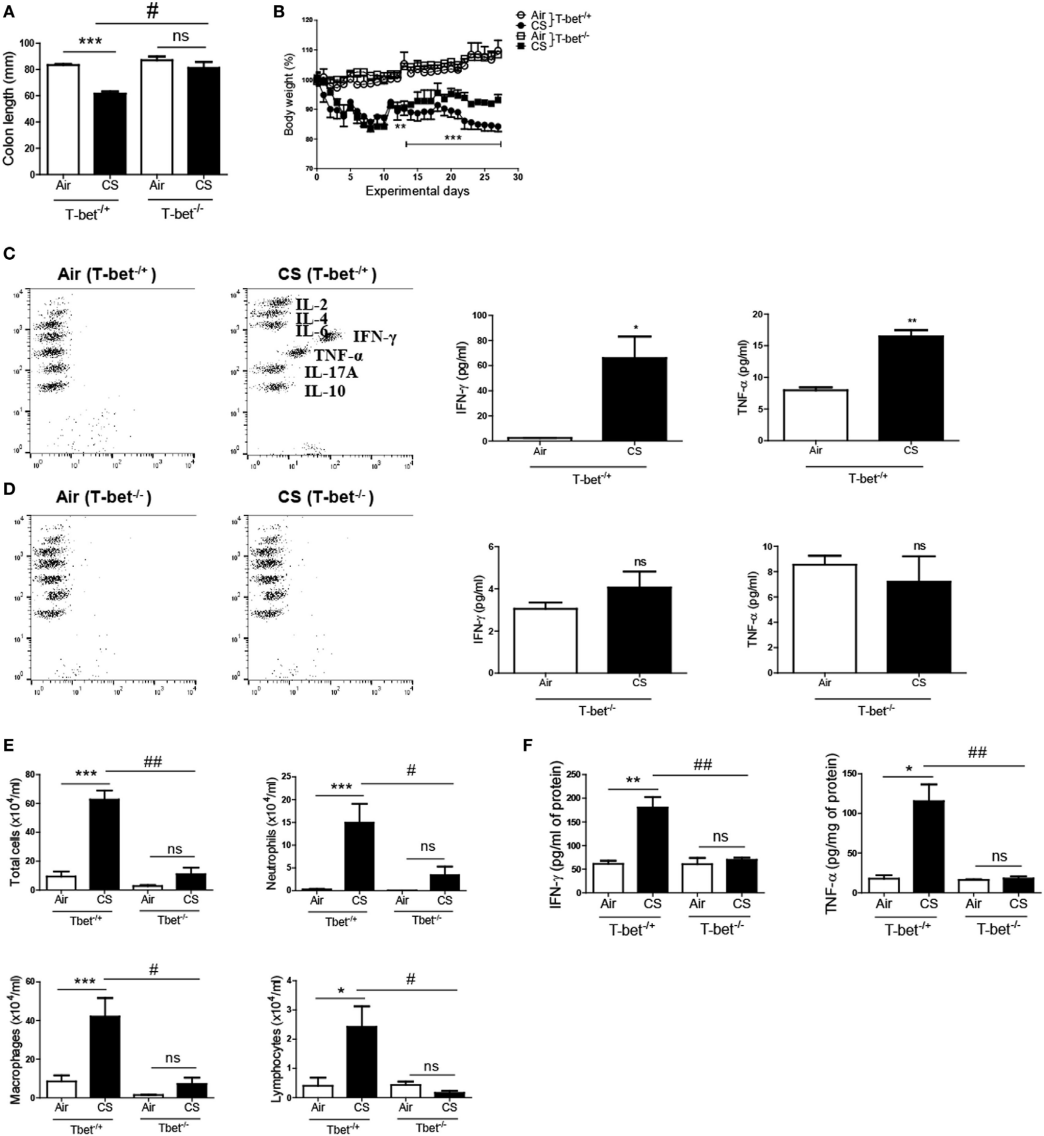
T-bet deficiency protects against the development of colitis and chronic lung inflammation induced by cigarette smoke (CS) exposure. T-bet^−/+^ and T-bet^−/−^ mice were exposed to CS for 4 weeks. **(A)** Colon length was measured 24 h after last CS exposure, and **(B)** the body weight was monitored for 4 weeks. **(C,D)** The concentration of IFN-γ and TNF-α was measured in ascending colon tissue homogenate using Th1, 2, and 17 cytometric bead array. On day 27, the mice were sacrificed, and bronchoalveolar lavage (BAL) fluid was collected. **(E)** The total cells, neutrophils, macrophages, and lymphocytes in the BAL fluid from the lung of mice were counted using Diff-Quick staining. **(F)** The concentrations of the Th1 cytokines IFN-γ and TNF-α in the BAL fluid were determined using enzyme-linked immunosorbent assay. Data are shown as the mean ± SEM, and *p* value was estimated by unpaired *t* test (***p < 0.001, **p < 0.01, and *p < 0.05, ^###^*p* < 0.001, ^##^*p* < 0.01, and ^#^*p* < 0.05, and not significant; ns).

T-bet is a Th1-specific transcription factor that regulates the production of IFN-γ in CD4^+^ T cells (27). As we observed the relevance of IFN-γ and the role of CD4^+^ T cells for CS-induced inflammation above, we exposed T-bet-deficient mice to CS for 4 weeks to assess the role of the Th1 response in CS exposure-induced colitis and lung inflammation. As shown in Figures 4A,B, T-bet^−/+^ mice showed a significant reduction in both the colon length and body weight following CS exposure; however, T-bet^−/−^ mice exhibited significant protection against CS exposure-induced changes in colon length and body weight. Additionally, T-bet^−/−^ mice showed unaltered expression of IFN-γ and TNF-α upon CS exposure, while T-bet^−/+^ mice showed significantly increased expression of IFN-γ and TNF-α following CS exposure in both colon and lung (Figures 4C,F). In T-bet^−/−^ mice, CS exposure also blocked the effect on infiltrated immune cell numbers and inflammatory cytokine concentrations (Figures 4D–F). These results indicate more clearly that CS-induced inflammatory responses are influenced by Th1 responses.

### IFN-γ Deficiency Inhibits the Development of Colitis and Chronic Lung Inflammation Induced by CS Exposure

**Figure 5:**
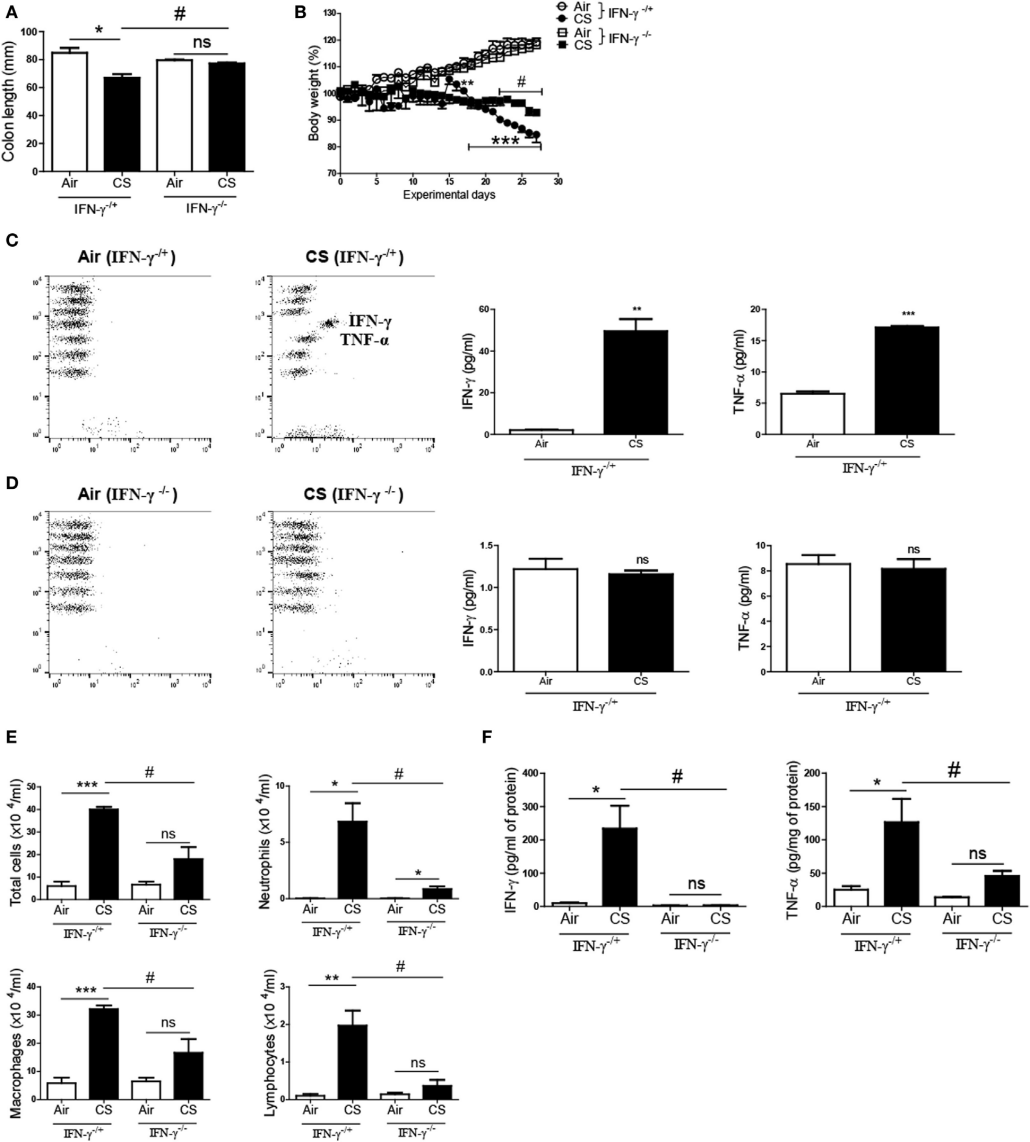
IFN-γ deficiency protects against the development of colitis and chronic lung inflammation induced by exposure to cigarette smoke (CS). IFN-γ^−/+^ and IFN-γ^−/−^ mice were exposed to CS for 4 weeks. **(A)** Colon length was measured 24 h after last CS exposure, and **(B)** the body weight was monitored for 4 weeks. **(C,D)** The concentrations of IFN-γ and TNF-α were measured in ascending colon tissue homogenate using Th1, 2, and 17 cytometric bead array. On day 27, the mice were sacrificed and bronchoalveolar lavage (BAL) fluid was collected. **(E)** The total cells, neutrophils, macrophages, and lymphocytes in the BAL fluid from the lung of mice were counted using Diff-Quick staining. **(F)** The concentrations of the Th1 cytokines IFN-γ and TNF-α in the BAL fluid were determined by using enzyme-linked immunosorbent assay. Data are shown as the mean ± SEM, and *p* value was estimated by unpaired *t* test (***p < 0.001, **p < 0.01, and *p < 0.05, ^#^*p* < 0.05, and not significant; ns).

To confirm whether CS-induced inflammatory responses are IFN-γ dependent in the colon, we exposed IFN-γ-deficient mice to CS. The CS exposure caused a noticeable decline in the colon length and the body weight in IFN-γ^−/+^ mice. Meanwhile, the CS-exposed IFN-γ^−/−^ mice demonstrated a significantly improved colon length compared to the CS-exposed IFN-γ^−/+^ mice (Figure 5A). Protection from body weight loss by IFN-γ deficiency was observed from day 22 (Figure 5B). CS exposure caused the IFN-γ^−/+^ mice to express highly increased concentrations of IFN-γ and TNF-α in the colon and lung, while no IFN-γ was detected in IFN-γ^−/−^ mice, as expected (Figures 5C,D). IFN-γ deficiency significantly inhibited immune cell infiltrations into the lung by CS exposure (Figure 5E). Interestingly, IFN-γ deficiency decreased CS exposure-induced secretion of TNF-α in the colon and also protected the lung from inflammation by CS exposure (Figures 5D–F).

### IFN-γ Plays a Substantial Role in Colitis, Which Is Induced by the AT of Lung Draining Lymph Node CD4^+^CD25^−^ T Cells in Colitis-Prone IL-10^−/−^ Mice

**Figure 6:**
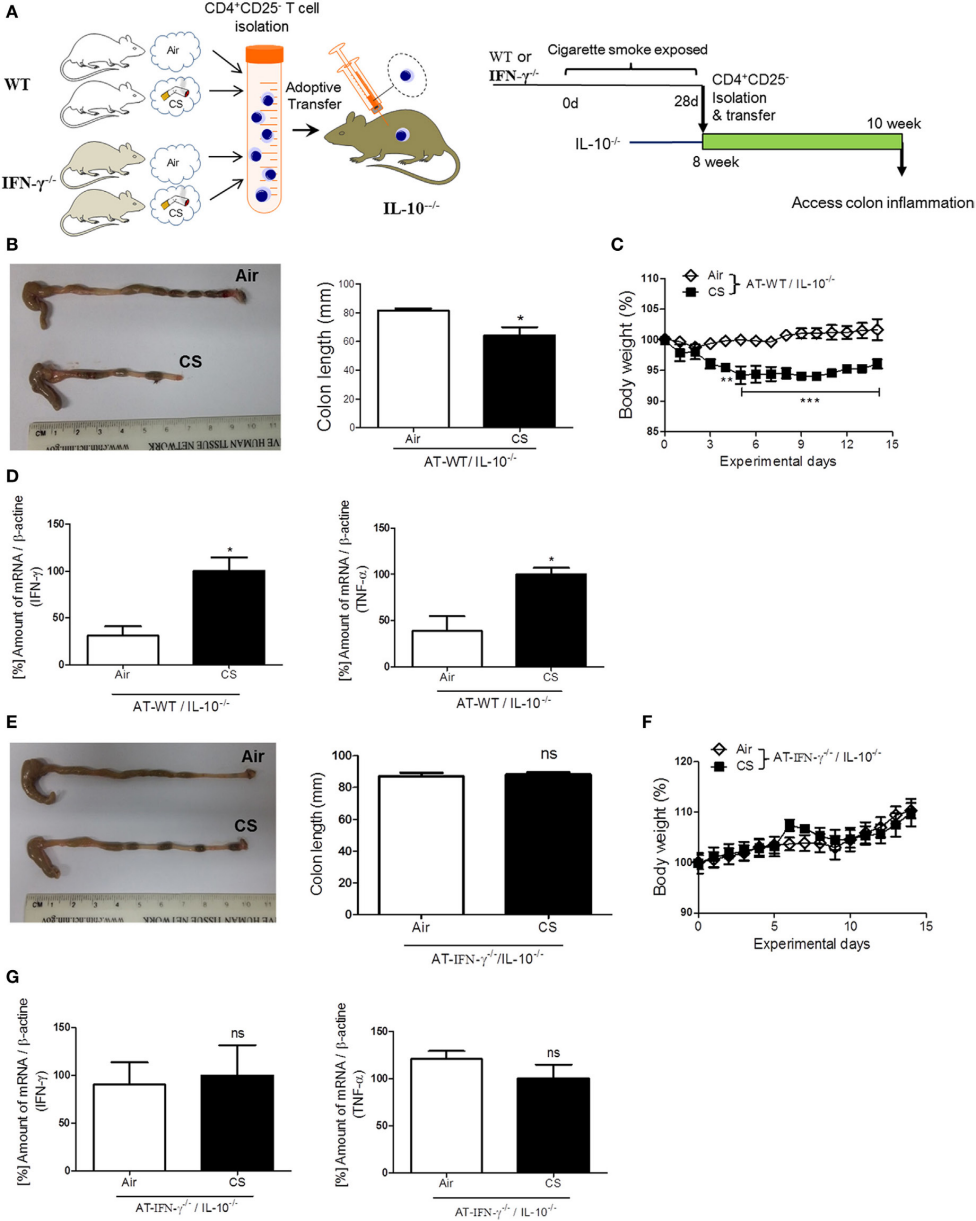
IFN-γ plays a substantial role in colitis induced by lung draining lymph node CD4^+^CD25^−^ T cells adoptive transfer (AT) in IL-10^−/−^ mice. WT and IFN-γ^−/−^ mice were exposed to cigarette smoke for 4 weeks. On day 27, CD4^+^CD25^−^ T cells were isolated from lung draining lymph node cells of mice and were adoptively transferred via i.v. into IL-10^−/−^ mice. On day 14 after AT, the mice were sacrificed. **(A)** Experimental design of colitis induction in IL-10^−/−^ mice. The schematic illustration was generated by modifying images purchased in the PPT Drawing Toolkits-BIOLOGY Bundle (Motifolio Inc., Elliocott City, MD, USA). **(B)** Representative images of whole colons (each group) and colon length. **(C)** Body weight changes after of CD4^+^CD25^−^ T cell AT. **(D)** Th1 cytokines IFN-γ and TNF-α mRNA expression in the colon was quantified by real-time PCR. **(E)** Representative images of whole colons (each group) and colon length. **(F)** Body weight changes after CD4^+^CD25^−^ T cell AT. **(G)** Th1 cytokines IFN-γ and TNF-α mRNA expression in the colon was quantified by real-time PCR. Data are shown as the mean ± SEM, and *p* value was estimated by unpaired *t* test (***p < 0.001, **p < 0.01, and *p < 0.05 and not significant; ns).

To confirm the role of IFN-γ in the induction of colitis via CS-stimulated CD4^+^ T cells, we performed an AT of lung draining lymph node CD4^+^CD25^−^ T cells from WT or IFN-γ^−/−^ mice to colitis-prone IL-10^−/−^ mice (Figure 6A) (28). Fourteen days later, after the AT of isolated lung draining lymph node CD4^+^CD25^−^ T cells from WT mice, body weight loss was observed in IL-10^−/−^ mice (Figure 6C), but the same cells adoptively transferred from IFN-γ^−/−^ mice did not cause body weight loss (Figure 6F). The AT of WT cells shortened colon length (Figure 6B) and increased IFN-γ and TNF-α expression (Figure 6D); however, the AT of IFN-γ-deficient cells did not cause those changes (Figures 6E,G), indicating that IFN-γ producing CS-exposed CD4^+^ T cells play a substantial role in inducing colitis. We also examined an expression of gut homing integrin, A4B7, in CD4^+^ T cells (29). When we check it in the blood and spleen, CS exposure increased the expression of A4B7 in CD4^+^ T cells (Figure S1D in Supplementary Material).

### Lung Draining Lymph Node CD4^+^CD25^−^ T Cells from CS Exposure Mice Migrate into Colon in IL-10^−/−^ Mice

**Figure 7:**
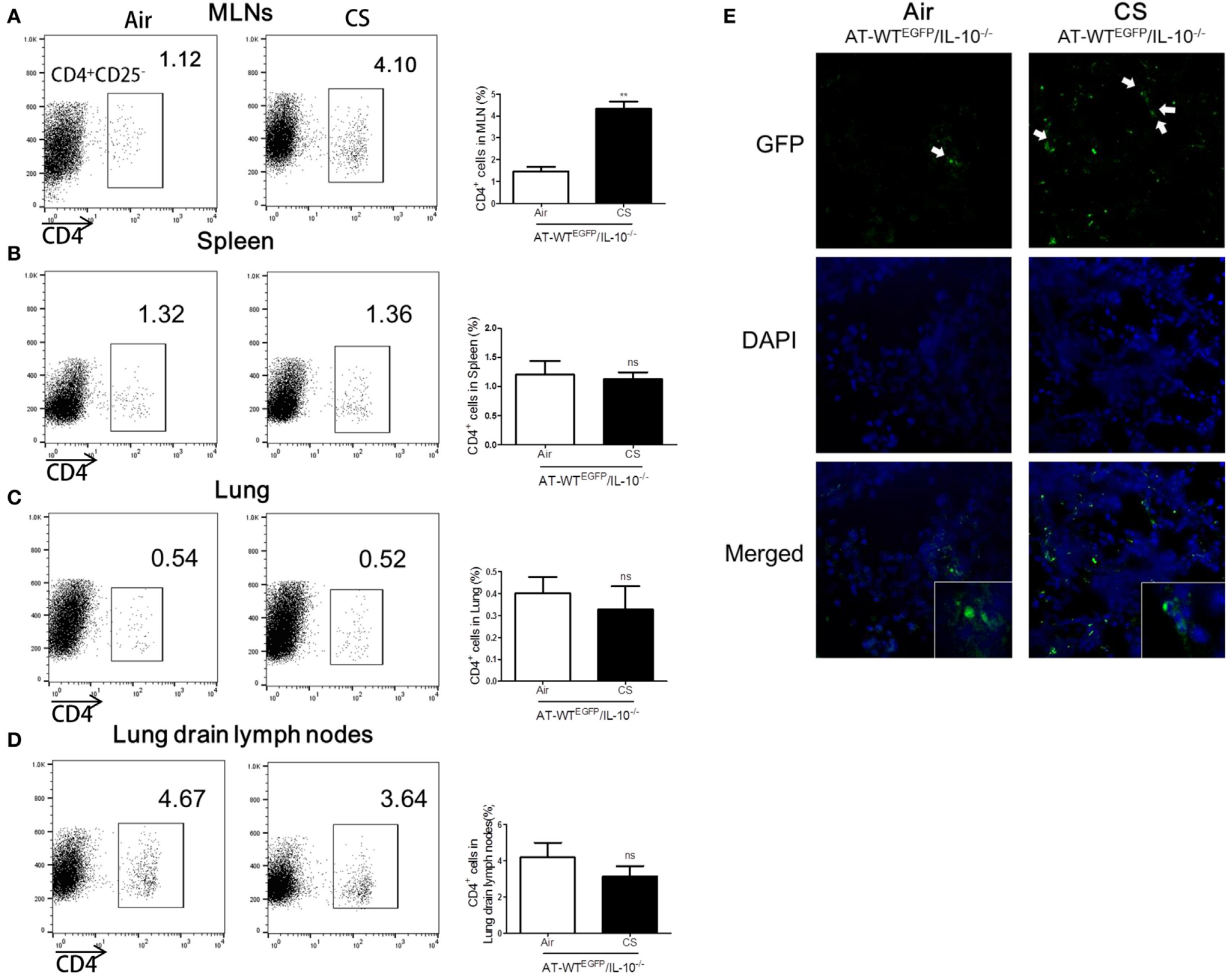
Cigarette smoke (CS) exposed CD4^+^CD25^−^ T cells from lung draining lymph node migrated into colon of IL-10^−/−^ mice. WT^EGFP^ mice were exposed to CS or air for 4 weeks. On day 27, CD4^+^CD25^−^ T cells were isolated from lung draining lymph node and adoptively transferred via i.v. into IL-10^−/−^ mice. Three days after adoptive transfer (AT), the mice were sacrificed and cell populations were analyzed from mesenteric lymph nodes (MLNs), spleen, lung, and lung draining lymph nodes. The population of CD4^+^ T cells in **(A)** the MLNs, **(B)** spleen, **(C)** lung, and **(D)** lung draining lymph node. **(E)** CD4^+EGFP^ T cells in the colon were observed by confocal microscopy. White arrows indicate GFP (CD4^+EGFP^ T cells). Data are shown as the mean ± SEM, and *p* value was estimated by unpaired *t* test (**p < 0.01 and not significant; ns).

To follow the migration of CS-stimulated CD4^+^ T cells into colon, we performed an AT of lung draining lymph node CD4^+^CD25^−^ T cells from WT^EGFP^ to IL-10^−/−^ mice. The IL-10^−/−^ mice which received the AT of CS-stimulated lung draining lymph CD4^+^CD25^−^ T cells observed a significant increase the population of CD4^+^ T cells in MLNs and colon compared to the Air group (Figures 7A,E). However, the other tissues including lung, lung draining lymph, and spleen did not show increase in CD4^+^ population (Figures 7B–D). Altogether, these data demonstrated that CS stimulates CD4^+^CD25^−^ T cells migration from lung draining lymph node to MLNs and colon in IL-10^−/−^ mice.

## Discussion

Cigarette smoking appears to play a major role in the pathogenesis of Crohn’s disease as well as pulmonary diseases (30). Epidemiological studies have provided strong evidence to confirm the increased incidence of Crohn’s disease in smokers. Multiple mechanisms may be responsible for the association of CS and colitis. CS reportedly increases the levels of pro-inflammatory cytokines, including IL-1, IL-6, IL-8, and TNF-α, and decreases those of anti-inflammatory cytokines such as IL-10 (31). Moreover, Petrescu et al. demonstrated a positive correlation between serum levels of TNF-α and CS exposure (32). We previously could also observe markedly increased inflammation in the colon following CS exposure (33). Notably, IFN-γ is highly expressed following CS exposure, although there was little change in the levels of IL-2, IL-4, IL-6, IL-10, and IL-17A in the colon.

IFN-γ-targeted therapy has been attempted and appears to be effective in the treatment of Crohn’s disease (34). It is well established that IFN-γ regulates intestinal epithelial homeostasis (35) and that IFN-γ-deficient mice exhibit a less severe progression of experimental colitis (36). Consistent with these reports, we showed here that the IFN-γ/Th1 cell-specific transcription factor *T-bet* is strongly involved in CS-induced colitis. CS exposure induced T-bet/IFN-γ-mediated colitis in normal mice. Indeed, the results are in agreement with the traditional notion that CS exposure boosts Crohn’s disease associated with a Th1 cytokine profile. The Th1/Th17 interaction is deeply associated with colitis (37, 38), IFN-γ inhibits the Th17 responses by blocking IL-23 expression in colitis (39). Additionally, the microbial flora affects the immune responses in the gut and CS seems to have an influence on the gastrointestinal microbiota (40). This microenvironmental difference may explain why IL-17A was detected in the lung but not in MLNs or colonic LP as well as in the ascending colon in our experiments.

Experiments using CD4^+^ T-cell or CD8^+^ T-cell depletion showed that the CD4^+^ T cell is a major cell subset causing CS-induced colitis, and other cells such as CD8^+^ T cells also have a role in this process. CS exposure failed to induce colitis in T-bet- or IFN-γ-knock-out mice, although there was a slight inflammatory tendency. These results suggest that CS exposure may induce colitis via Th1 responses, but the mechanisms are incompletely understood, with the exception of the Th1-mediated mechanism underlying the immune imbalance in colitis and the involvement of CS. In addition, it is still unclear what the relation between CS-induced colitis and ulcerative colitis is. In a previous study, CS showed a protective effect against ulcerative colitis (41) and is thought to have protective effects against the development of ulcerative colitis associated with Th2-mediated disease (42). According our results, it is possible that CS may protect from ulcerative colitis via the downregulation of Th2 responses *via* Th1 responses.

Previous animal studies investigating the effect of CS exposure on gut have shown conflicting results. Zuo et al. showed that CS is associated with intestinal barrier dysfunction in the small intestine but not in the colon (43). They used BALB/C, prototypical Th2-type mouse strain while we used C57BL/6 prototypical Th1-type strain. Different T-cell differentiation between these mouse strains may affect the development of inflammation in the small intestine and colon (44). Montbarbon et al. reported that CS exposure for 3 weeks did not induced colitis but increased only several cytokines including IL-10 and IL-13 (45). They claimed that CS exposure recruit invariant natural killer T cells in the colon, and these cells can protect tissue from DSS induced colonic inflammation. However, they also showed that CS exposure increased mRNA expression of inflammatory cytokines including TNF, IFN-γ, IL-17, and IL-21 in Ja18^−/−^ mice and escalated clinical symptoms of DSS-induced colitis in CD1d^−/−^ mice. As it is hard with CS alone to induce severe inflammation on gut, many studies have used chemically induced colitis models to study the impact of CS exposure on gut inflammation (46). However, in our experiment, CS alone could induce histological change of colon and increased expression of inflammatory cytokines including IFN-γ and TNF-α. The dissimilarities between researches may be explained by differences in the methods of CS simulation.

Verschuere et al. showed that CS exposure alone increases apoptosis in the follicle-associated epithelium and is associated with immune cells including dendritic cells, CD4^+^ T cells, and CD8^+^ T cells accumulation in Peyer’s patches. They examined the effect on the gut-associated lymphoid tissue of the small intestine, Peyer’s patches in particular, because of the known relationship between CS and Crohn’s ileitis (47).

In conclusion, we unveiled the role of IFN-γ^+^ CD4^+^ T cells on CS-mediated colitis in animal model. Our findings identify a novel mechanism of CS-induced colitis: the mediation of immune activation via T-bet/IFN-γ-mediated CD4^+^ T cells. IFN-γ plays a substantial role in colitis induced by CS exposure. An understanding of the T-bet/IFN-γ-mediated CD4^+^ T cell changes in the colon extends our understanding of CS-induced pathology, which may result in the development of novel therapeutic strategies to treat Crohn’s disease.

## Ethics Statement

All of the experiments were performed in accordance with the approved animal protocols and guidelines established by Kyung Hee University [KHUASP (SE)-12-015].

## Author Contributions

KJ performed the majority of experiments. GL contributed to the interpretation of data and wrote the manuscript. DS, CL, WK, and SL contributed to the acquisition of data. JK and HB designed the research and analyzed the data. All authors reviewed and approved the manuscript.

## Conflict of Interest Statement

The authors declare that the research was conducted in the absence of any commercial or financial relationships that could be construed as a potential conflict of interest.

## Supplementary Material

Figure S1 **(A)** The percentage of various immune cell in colonic lamina propria. **(B)** Fecal blood test result after 2 weeks CS exposure. **(C)** The percentage of IFN-γ producing cells in CD4^+^ T cells, CD8^+^ T cells, and NK cells. After 2 weeks of CS exposure, splenocytes were incubated for 5 h with PMA, Ionomycin, and protein transport inhibitor. **(D)** A4B7 expression in CD4^+^ T cells. After 2 weeks of CS exposure, expression of A4B7 was measured in the blood and spleen (***p* < 0.01 and **p* < 0.05 and not significant; ns).
