# Supplementary figures and images for: Cigarette Smoking Triggers Colitis by IFN-γ+ CD4+ T Cells
Source: Front Immunol. 2017 Oct 31;8:1344. doi: 10.3389/fimmu.2017.01344 (PMC5671659; doi:10.3389/fimmu.2017.01344)

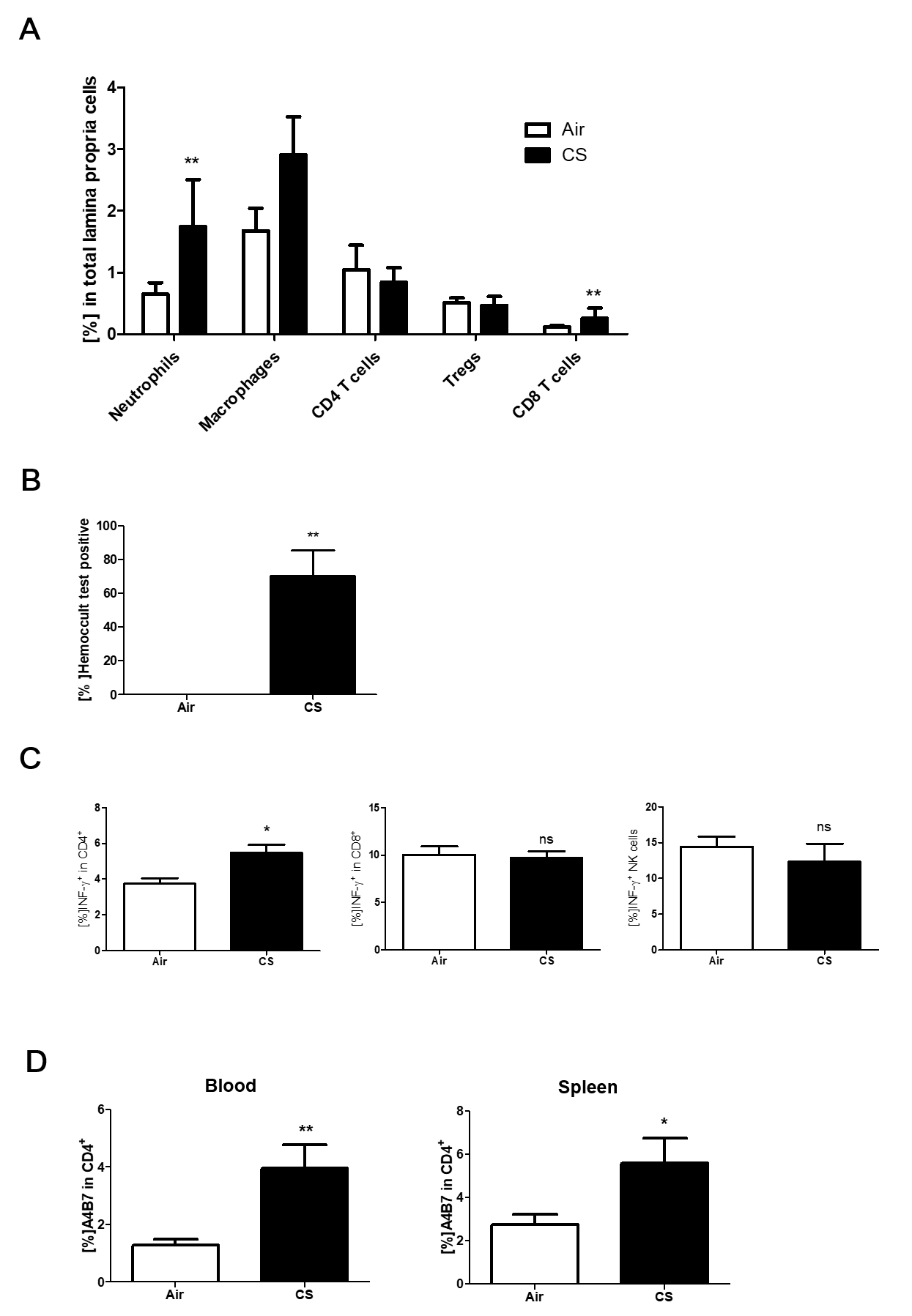

Supplement: Figure S1 — (A) The percentage of various immune cell in colonic lamina propria. (B) Fecal blood test result after 2 weeks CS exposure. (C) The percentage of IFN-γ producing cells in CD4+ T cells, CD8+ T cells, and NK cells. After 2 weeks of CS exposure, splenocytes were incubated for 5 h with PMA, Ionomycin, and protein transport inhibitor. (D) A4B7 expression in CD4+ T cells. After 2 weeks of CS exposure, expression of A4B7 was measured in the blood and spleen (**p < 0.01 and *p < 0.05 and not significant; ns). [file Image_1.TIF]
